# A multi-site single blind clinical study to compare the effects of prolonged exposure, eye movement desensitization and reprocessing and waiting list on patients with a current diagnosis of psychosis and co morbid post traumatic stress disorder: study protocol for the randomized controlled trial Treating Trauma in Psychosis

**DOI:** 10.1186/1745-6215-14-151

**Published:** 2013-05-23

**Authors:** Paul AJM de Bont, David PG van den Berg, Berber M van der Vleugel, Carlijn de Roos, Cornelis L Mulder, Eni S Becker, Ad de Jongh, Mark van der Gaag, Agnes van Minnen

**Affiliations:** 1 Mental Health Organization (MHO) GGZ Oost Brabant Land van Cuijk en Noord Limburg, Bilderbeekstraat 44, Boxmeer, 5831 CX, The Netherlands; 2 Behavioural Science Institute, NijCare, Radboud University, Montessorilaan 3, P.O. Box 9104, Nijmegen, 6525 HR, The Netherlands; 3 Parnassia Psychiatric Institute, Prinsegracht 63, Den Haag, 2512 EX, The Netherlands; 4 Department of Clinical Psychology, VU University Amsterdam and EMGO Institute for Health and Care Research, Van der Boechorststraat 1, Amsterdam, 1081 BT, the Netherlands; 5 MHO GGZ Noord-Holland Noord, Oude Hoeverweg 10, Alkmaar, 1816 BT, The Netherlands; 6 MHO Rivierduinen, Schuttersveld 9, P.O. Box 2211, Leiden, 2316 XG, the Netherlands; 7 Department of Psychiatry, and BavoEuropoort, University Medical Center Rotterdam, Dr. Molewaterplein 50, Rotterdam, 3015 GE, The Netherlands; 8 Department of Behavioral Sciences, Academic Centre for Dentistry Amsterdam (ACTA), Gustav Mahler Laan 3004, Amsterdam, 1081 LA, The Netherlands; 9 Department of Behavioral Sciences, Academic Centre for Dentistry Amsterdam (ACTA), VU University Amsterdam, Gustav Mahler Laan 3004, Amsterdam, 1081 LA, The Netherlands; 10 School of Health Sciences, Salford University, The Crescent, Salford, M5 4WT, United Kingdom; 11 MHO ‘Pro Persona’, Centre for Anxiety Disorders Overwaal, Pastoor van Laakstraat 48, 6663 CB, Lent, The Netherlands

**Keywords:** Psychosis, PTSD, Trauma, Schizophrenia, Hallucinations, Working memory, Prolonged exposure, EMDR, Economic evaluation, Moderators, Mediators

## Abstract

**Background:**

Trauma contributes to psychosis and in psychotic disorders post-traumatic stress disorder (PTSD) is often a comorbid disorder. A problem is that PTSD is underdiagnosed and undertreated in people with psychotic disorders. This study’s primary goal is to examine the efficacy and safety of prolonged exposure and eye movement desensitization and reprocessing (EMDR) for PTSD in patients with both psychotic disorders and PTSD, as compared to a waiting list. Secondly, the effects of both treatments are determined on (a) symptoms of psychosis, in particular verbal hallucinations, (b) depression and social performance, and (c) economic costs. Thirdly, goals concern links between trauma exposure and psychotic symptomatology and the prevalence of exposure to traumatic events, and of PTSD. Fourthly predictors, moderators, and mediators for treatment success will be explored. These include cognitions and experiences concerning treatment harm, credibility and burden in both participants and therapists.

**Methods/Design:**

A short PTSD-screener assesses the possible presence of PTSD in adult patients (21- to 65- years old) with psychotic disorders, while the Clinician Administered PTSD Scale interview will be used for the diagnosis of current PTSD. The M.I.N.I. Plus interview will be used for diagnosing lifetime psychotic disorders and mood disorders with psychotic features. The purpose is to include consenting participants (N = 240) in a multi-site single blind randomized clinical trial. Patients will be allocated to one of three treatment conditions (N = 80 each): prolonged exposure or EMDR (both consisting of eight weekly sessions of 90 minutes each) or a six-month waiting list. All participants are subjected to blind assessments at pre-treatment, twomonths post treatment, and six monthspost treatment. In addition, participants in the experimental conditions will have assessments at mid treatment and at 12 months follow-up.

**Discussion:**

The results from the post treatment measurement can be considered strong empirical indicators of the safety and effectiveness of prolonged exposure and EMDR. The six-month and twelve-month follow-up data have the potential of reliably providing documentation of the long-term effects of both treatments on the various outcome variables. Data from pre-treatment and midtreatment can be used to reveal possible pathways of change.

**Trial registration:**

Trial registration: ISRCTN79584912

## Background

### Links between trauma, PTSD and psychosis

Of patients who have ever experienced psychotic episodes, 50% to 98% report having been exposed to one or more traumatic events in their lives [1]. As a result, the prevalence of post traumatic stress disorder (PTSD) in people with psychotic disorders ranges from 12% to 29% [2,3]. This can be considered high compared to estimated prevalence rates in the general population, which range from 0.4% to 3.5% [4-6].

In a meta-analytical study evidence was found that major adversities in childhood (before the 18th year of life) increase the risk of psychosis by 2.8 times on average [7]. A dose–response relationship was found in almost all studies: the more severe the trauma, the greater the risk of developing psychosis. Childhood trauma as a major risk factor probably adds 33% to the onset of psychosis in society [7]. Significant associations between all types of childhood adversities (except the loss of a parent) and symptoms of paranoia and auditory hallucinations have been reported [7,8]. Childhood sexual abuse is associated with hallucinations (odds ratio (OR) 8.9, confidence interval (CI) = 1.86 to 42.44), and being brought up in institutional care is specifically associated with paranoia (OR = 11.08, CI = 3.26 to 37.62 [8].

Traumatic events, PTSD and psychosis appear to have several interactions. The presence of a comorbid PTSD has been found to have a negative impact on the course and prognosis of the psychotic disorder [9], and the combination of psychosis and PTSD appears to be associated with poorer social functioning and greater risk of relapsing in psychosis [9-11]. There are also indications that the content of hallucinations may fully match that of experienced traumas (12.5%) or the themes of hallucinations can be very similar to that of experienced traumas (45%) [12]. The occurrence of re-experiencing symptoms of PTSD is strongly associated with a predisposition to hallucinations. Negative beliefs about self and others have been found to be associated with a predisposition to paranoia [13].

There is also evidence to suggest that experiencing a psychotic episode, and experiencing problems with the health care system, may potentially be experienced as traumatic events and may result in PTSD [13-15].

Considering these findings and being aware that PTSD in general is associated with forms of non-effective coping, more abuse of alcohol and drugs, negative self-esteem, negative expectations of other people, and a greater risk of exposure to future potentially traumatic events [16], it becomes clear that trauma exposure is associated with impairment and major health problems in patients with psychosis, creating a burden for both patients and society [17,18].

### Clinical attitudes and practice

In clinical practice, trauma exposure, PTSD and their links to psychosis do not seem to get that much attention. Traumatic experiences and the diagnosis of PTSD appear to be underreported in the charts of patients with a severe mental illness or psychotic disorder [19,20]. In addition, clinicians are reluctant to address trauma histories in patients with psychosis [21,22]. They will not easily offer therapy, especially not exposure therapy, to overcome PTSD symptoms; instead, they suggest establishing trust and rapport with patients first, and giving patients a sense of mastery, before starting to explore traumatic experiences the patients might have had [23]. Many clinicians seem to fear that trauma treatment with this patient group will lead to symptom exacerbation, crisis, hospitalization, self-harm and suicidal behavior, or rapid changes in medication. They presume that before a PTSD treatment can be safely conducted, stabilizing interventions are necessary [23,24].

### Studies examining PTSD treatment efficacy

Meta-analytic studies of clinical trials show that, generally speaking, trauma focused cognitive behavioral therapy (TF-CBT), such as prolonged exposure (PE), and eye movement desensitization and reprocessing (EMDR) are safe and effective. These treatments should be the first-line psychological treatment for PTSD [25-27]. Unfortunately, people with a psychotic disorder are often excluded from PTSD treatment efficacy research [28,29]. Therefore, not much is known about the generalizability of PE or EMDR efficacy to people with psychotic disorders [30]. This question of applicability of PE and EMDR in patients with psychosis is the starting point of the present study protocol.

A number of studies have already addressed this issue. In a randomized controlled trial (RCT), the effects of Cognitive Behavior Therapy (CBT) were investigated in 108 participants with severe mental illness, of which 14% (N =17) had a diagnosis of schizophrenic or schizoaffective disorder [31]. The protocol consisted of 12 to 16 sessions of combined CBT interventions (a crisis plan, psycho-education, breathing exercise, cognitive restructuring). CBT proved to be effective in reducing PTSD symptoms and was safe. In an open study, the effect of PE in people with psychosis was studied [32]. Participants (N = 20) were given fourteen preparatory sessions and eight PE sessions. The drop out was high during preparatory sessions and this phase did not result in a decline of symptoms. A high compliance, no adverse events and a significant reduction of PTSD symptoms, during and after PE, were, however, noted. These results suggest that PE is effective and safe and preparation for PE may be unnecessary.

Recently, our research group conducted two small feasibility trials. The first, an open study (N = 27), used EMDR for PTSD in people with a life time history of psychosis [33]. The second study used EMDR (N = 5) and PE (N = 5) in a multiple baseline controlled design [34]. Results showed that both treatments were effective in reducing PTSD symptoms. Van den Berg and Van der Gaag [33] found significant reductions in end-state PTSD, symptom severity of PTSD, delusions, hallucinations, depression and anxiety and an increase in self-esteem. De Bont and colleagues [34] found significant reductions of PTSD and of general psychopathology and distress. In both studies no serious adverse events or symptom exacerbations were found. Both treatments were well accepted by participants and attrition was low. Taken together, results from pilot studies suggest that trauma-focused treatments, such as PE and EMDR, can be effectively and safely applied to patients with psychotic disorders, but well-controlled studies are lacking. To fill this gap, we set up the present study, the Treating Trauma in Psychosis (T.TIP) study.

## Objectives

### Primary objective –effects of treatment on PTSD symptoms and safety

In a sample of patients who receive treatment as usual (TAU) for a current diagnosis of psychosis this study examines: a) the efficacy of PE versus waiting list, and of EMDR versus waiting list, on the symptomatology of a comorbid PTSD; and b) the safety of PE versus waiting list and EMDR versus waiting list in the treatment of PTSD.

### Secondary objectives - effects of treatment on psychopathology and cost effectiveness

Other objectives were to determine the efficacy of PE versus waiting list and of EMDR versus waiting list on the symptomatology of psychosis. In this context the experience sampling method (ESM) [35] will be applied to examine the effects of treatment on temporal associations between intrusions, daily events and activities, and verbal hallucinations, depression,social functioning andpost traumatic cognitions.An economic evaluation will determine the cost effectiveness of the PTSD treatments.

### Tertiary objectives - links between trauma exposure, PTSD and psychosis

An important objective is to assess, in the psychosis sample, exposure to traumatic events, to assess and calculate the risk of having a comorbid PTSD, and to diagnose comorbid PTSD. In this context, the diagnostic accuracy of the Trauma Screening Questionnaire [36], in the population of patients with psychotic disorder, will be examined.

The present T.TIP study will address the question as to whether certain types and severities of trauma exposure are associated with specific phenomenological aspects of psychotic symptoms [7,8,37,38]. Specifically, the study aims to explore the links between traumatic events, PTSD and verbal hallucinations [12,33].

### Quaternary objectives – mediators, moderators and predictors of treatment outcome

In the intervention study we will conduct additional research to explore mechanisms of changes in the treatment outcome variables. Variables that are potential predictors, mediators and moderators will be examined, such as: a) bullying [7,8]; b) tonic immobility [39-41]; c) the capacity of participants’ working memory [42,43]; d) credibility of treatment and burden of treatment [44,45]. Both participants and therapists will have cognitions about treatment that may affect treatment outcome. Therefore assessments will be made of harm expectancy and harm experience, expected burden and experienced burden and credibility of treatment; e) demographic and trauma characteristics; f) self esteem [46-48]; g) personal beliefs about illness [49,50]; h) post traumatic cognitions [51]; i) cognitive biases [52-54]; j) cognitions about voices; k) memory characteristics [55-58]; and l) social support during treatment [59,60].

## Methods/Design

### Design

The T.TIP study is a single blind randomized controlled trial with three arms: PE, EMDR and a waiting list. The three groups are compared at pre-treatment (T0), at post treatment at two months (T2) and at six months (T6), on a set of variables that are linked to the various research questions. Mediator and moderator variables are assessed again at midtreatment (T1). A 12-month treatment intervention follow-up measurement (T12) will give indications whether treatment effects endure. The waiting list condition will receive the treatment of choice after sixmonths (T6).

The assessing research assistants are blind to the participants’ research condition.

The design of this study was approved by the Medical Ethics Committee of the medical centre of the VU University and is registered as N:36649.029.12.

### Participants

Patients, 18- to 65-years old, with a chart diagnosis of psychotic disorder or a mood disorder with psychotic features according to the Diagnostic and Statistical Manual of Mental Disorders, fourth edition text revsion(DSM-IV-TR) are recruited from outpatient services of thirteen mental health organizations in the Netherlands. The trial process starts with a short self-report screening. During the screening, participants report on traumatic events that fulfill DSM-IV-TR PTSD criterion A1 and associated PTSD symptoms using the 10 item Trauma Screening Questionnaire (TSQ) [36]. If participants report traumatic events and score above the TSQ’s cut-off criterion (*x* = ≥6), and give informed consent for further interviewing, an appointment will be made for the inclusion interview.

### Inclusion and exclusion

The criteria for inclusion in the intervention study are: a) age between 18 and 65 years; b) a lifetime history of a psychotic disorder or a mood disorder with psychotic features (M.I.N.I. plus); c) meeting DSM-IV-TR diagnostic criteria for PTSD (Clinician Administered PTSD Scale (CAPS)).

The criteria for exclusion are: a) high suicidality, operationalized as the combination of having a high suicidality score on the M.I.N.I. with the last suicide attempt within the past six months and a depression score on the Beck Depression Inventory-II (BDI-II) of 35 or higher; b) changes in medication (mood regulators, antipsychotics) within two months prior to the study; c) insufficient competence in the Dutch language; d) severe intellectual impairment, defined as an estimated IQ below 70 (mental retardation); e) not being able to travel to and from the place of assessment and therapy; and f) participant is in seclusion or admitted to a closed ward.

**Figure 1:**
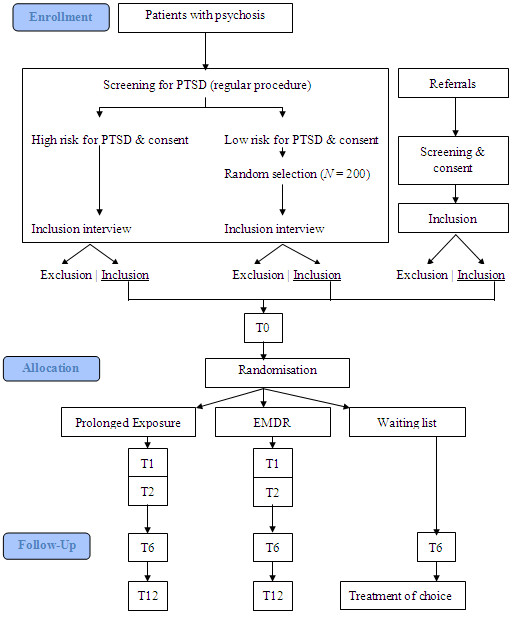
Flow Diagram T.TIP.

See Figure 1, the T.TIP flowchart. All mental health teams that signed up for cooperation in the T.TIP study carry out the standard procedure for screening of all patients suffering from psychosis. After that, the flow of consenting and screened participants into the study has three pathways: (1) ‘regular’ inclusion, meaning that each patient demonstrating a high risk for PTSD (TSQ ≥6) will be invited for an inclusion interview; (2) ‘low TSQ’ inclusion, meaning that a random sample of patients across the teams (N = 200) demonstrating a low risk of having a PTSD (TSQ ≤5) will be invited for a ‘low TSQ’-inclusion interview; and (3) ‘incidental referrals’-inclusion.

The inclusion interview conducted by the research assistant (RA) will be the same for all three categories ‘regular’, ‘low TSQ’ and ‘incidental referrals’: (a) three sections of the M.I.N.I.-Plus interview for assessing psychotic disorders, mood disorders with psychotic features and a risk analysis regarding acute suicidality; (b) the CAPS [61] for assessing a PTSD diagnosis; and (c) the BDI-II [62] for assessing the severity of depressive symptoms.

‘Regular’ participants who meet the inclusion criteria and give consent go to the randomization phase. ‘Low TSQ’-participants who appear to meet all criteria for inclusion (including a PTSD despite their low TSQ screening score) may also enter the randomization phase. The third group is that of incidental ‘referrals’. Referred participants will not be recruited using the standard screening procedures in cooperating teams. Instead, psychiatrists, psychotherapists, caseworkers or patients from other teams will be offered the opportunity to refer for screening and possible entry in the intervention study. They may do so, if thereare indications the patient may have psychosis and PTSD and may benefit from participation. The diagnostic data of referred participants will be excluded from some of the study’s analyses, but these will be part of all treatment outcome analyses.

### Randomization

All patients meeting the inclusion criteria are asked for written informed consent to participate in the intervention study. Consenting participants receive the pre-treatment (T0) measurements. (Note that for patients with auditory hallucinations six days of T0 measurements on auditory verbal hallucinations are added, using ESM; see Instruments). After T0, participants are randomized to PE, EMDR or the waiting list. A group of fifteen random assignments for each participating therapist will be made. These groups are made using the scientific randomization program on the Internet (http://www.randomizer.org) by the independent randomization bureau of the Parnassia Psychiatric Institute. Each block has five assignments for each condition: PE or EMDR or waiting list. Thus, each therapist treats five people with PE and five people with EMDR. After six months, the therapist will offer the five people from the waiting list the therapy of their choice.

### Power and sample size calculation

An F-test over three groups with a medium effect-size of treatment compared to waiting list, alpha .05 and power .80 needs 159 participants. Attrition is estimated conservatively to be high at 33%. The needed number of participants is 240. We will use 3 x 80 participants.

To minimize missing values: (a) research assistants will use letters, text messages, telephone calls, and contacts with participants' case workers for inviting participants and for reminding them about their assessment appointments; (b) therapists are reminded by mail and in supervision to conduct their assessments, be it assessments within sessions or at T1, T2 and T6; and (c) therapists are encouraged by mail to remind and support the participant to go to the assessment appointments with the RA at T2, T6 and T12.

All the participants will receive financial compensation of twenty-five euros for every single measurement at T0, T2, T6 and T12, regardless of whether or not the participant drops out of treatment. The compensation will be paid at the planned end of participation (T6 for waiting list participants, T12 for participants in the treatment condition).

### Interventions

#### Treatment as usual (TAU)

Participants in all three conditions PE, EMDR and waiting list receive TAU, consisting of antipsychotic medication, and treatment and/or supportive counseling by therapists, caseworkers or coaches (for example, Individual Placement and Support). TAU is considered to be equal in the three conditions as a result of the randomization procedure used (see Randomization).

### Prolonged exposure (PE)

PE therapy is a psychotherapeutic approach that reduces PTSD symptom-severity by systematically exposing the subject,in both imaginary and *in vivo* ways, to previously avoided traumatic internal and external stimuli. [63].Thetrauma-related fear memory network is activated by PE and psychopathological cognitions may be changed during PE [64]. PE consists of eight sessions of 90 minutes.

In the first treatment session, a case conceptualization is made in order to plan the treatment sessions. The therapist receives information from the Interview for Traumatic Events in Childhood (ITEC) [65], an interview that is part of T0. The ITEC captures categories (for example, sexual abuse, physical abuse) of experienced traumatic events. In addition, the intensity of re-experiencing symptoms of each traumatic event is assessed. Based on the ITEC, the therapist draws up a preliminary, formatted PE case conceptualization. In the first treatment session, the therapist will discuss and complete the case conceptualization together with the patient. This results in a hierarchy of the most relevant traumatic memories in order of significance with regard to the symptoms of PTSD. A highly qualified supervisor (AvM) reviews each case conceptualization.

In the first session, the therapist will explain the treatment rationale to the participant. Imaginary PE is carried out from the second treatment session: the confrontation with avoided anxiety provoking memories, during 45 to 60 minutes per session. The participant imaginarily relives the traumatic events. The therapist prompts the participant to tell details about the memory and to relive the memories very vividly, as if it was happening here and now. The participant is instructed to reveal sensory details of the traumatic events, and to talk in the present tense and from a personal first person perspective. The therapist helps the participant to expose himself or herself to the most fearful parts of the memory, the so-called hot spots. Subjective Units of Distress (SUDs, range 0 to 100) reflecting the levels of distress are monitored during the PE sessions. Each session is recorded and stored with a voice recorder. At home the participant listens to the complete recording five days a week, scoring the SUDs prior to, during and after listening. At session three exposure *in vivo* is added. A list is made of avoided trauma related stimuli. As homework, the participant sets out to confront these situations *in vivo* (including: on the internet). Again distress levels (SUDs) prior to, during, and after *in vivo* exposure are monitored.

### Eye movement desensitization and reprocessing (EMDR)

EMDR is a psychotherapeutic approach, in which memory representations of traumatic life experiences are processed in order to change dysfunctional beliefs about self or others that originated from damaging traumatic experiences [66,67]. EMDR consist of eight sessions of 90 minutes.

As in PE, in the first treatment session a case conceptualization is made in order to plan the treatment sessions. The therapist receives information from the ITEC. The ITEC captures categories (for example, sexual abuse, physical abuse) of experienced traumatic events. In addition, the intensity of re-experiencing symptoms of each traumatic event is assessed. Based on the ITEC, the therapist draws up a preliminary, formatted EMDR case conceptualization. In the first treatment session, the therapist will discuss and complete the case conceptualization together with the patient. This results in a hierarchy of the most relevant traumatic memories in order of significance with regard to the symptoms of PTSD. A highly qualified supervisor (CdR) reviews each case conceptualization.

After acceptance, memories are subsequently processed following the Dutch translation [68,69] of the basic EMDR protocol [67]. The participant is asked to focus on the currently most distressing image of a memory in a multi-modal manner, including image, thought (an experienced negative cognition (NC) and a more healthy positive cognition (PC)), emotion, physical sensation and level of tension (SUD). Processing of the memory starts when the therapist asks the patient to hold the target image in mind while concentrating on a distracting stimulus (the finger of the therapist eliciting eye movements) for about 30 seconds. The patient reports briefly what comes to mind and is guided by the clinician to refocus on that element while focusing on the distracting stimulus. This continues until no more associations come up and the disturbance level associated with the target memory (SUD) drops to zero. Then the therapist guides the participant in installing the PC to a maximum validity, that is a Validity of Cognition (VOC) score seven. The participant identifies any residual disturbing sensations and, if present, the memory will be processed again in the same manner as described above, until the SUD is zero and the VOC PC is seven. The therapist facilitates a positive closure to the session. In the next session a re-evaluation takes place in which the participant comments on previously processed targets as a basis for further intervention.

### Early completions

A participant will be considered an early completer of treatment when (a) his or her score on the PTSD Symptoms Scale – Self Report (PSS-SR) is lower than 10 on two consecutive occasions, and if (b) the SUDs from all situations that are part of the case conceptualization are reduced to zero.

### Measurements

Subjects participate in the study for 12 months. At T0, the RA assesses baseline measurements of all primary, secondary, tertiary and quaternary variables. At T1, at midtreatment, participants in treatment conditions are assessed for their scores on the moderator and mediator variables by self-report questionnaires handed out by the therapist. At T2, the end of treatment, the RA assesses treatment outcome variables. T6 is the first follow-up measurement, and this is the starting point for treatment for the waiting list group. T12 is the 12-month follow up measurement for the PE and EMDR group.

For participants in the treatment conditions PE and EMDR some assessments are planned within sessions: PTSD symptom severity, therapy burden, harm expectancy (pre-session) and experienced harm (post-session), adverse events (pre- and post-session), credibility of treatment and memory characteristics. Note that therapists are also assessed independently from the participants for perceived and experienced treatment burden credibility and harm, before, during and after treatment.

### Instruments

#### Measurements – inclusion

The M.I.N.I.-International Neuropsychiatric Interview-Plus (M.I.N.I.-Plus) [70-72] is used as an inclusion tool. The M.I.N.I.-Plus sections for psychotic and mood disorders will be used to assess lifetime psychotic disorder or mood disorder with psychotic features at recruitment. The M.I.N.I.-Plus is a short diagnostic interview schedule that can be easily incorporated into routine clinical interviews and is well accepted by patients [73]. The M.I.N.I.-Plus may be used in research [74]; it is fit to classify psychotic and mood disorders [74,75]. According to the C.I.D.I. [70] the Kappa coefficient, sensitivity and specificity of the M.I.N.I. were good (>0.70) or very good (>0.80) for most diagnoses including psychotic and mood disorders. Inter-rater and test-retest reliability were good (>0.70). Validation against the Structured Clinical Interview for DSM-IV (SCID-P) supported the validity and reliability of the M.I.N.I. [71].

As pointed out in the section Inclusion, the original M.I.N.I. module of suicidality was used as part of the algorithm used to exclude patients.

### Measurements –effects of treatment on PTSD symptoms and safety

See Table 1, Primary outcome measurements.

The CAPS [61] is the primary outcome measure. The CAPS assesses the presence or absence of PTSD diagnosis and the frequency and intensity of the clinician's rated PTSD symptoms. The CAPS provides ratings of the frequency and intensity of each of the 17 DSM-IV–TR based PTSD symptoms on 0 to 4 Likert-type scales, thereby allowing for a maximal score of 8 for each symptom and a total-score range from 0 to 136. The CAPS is considered the gold standard to diagnose posttraumatic stress disorder as defined in the DSM-IV-TR and to establish its severity. A review of the empirical literature on psychometric properties of the CAPS [76] indicates that the CAPS has excellent reliability (>0.90), yielding consistent scores across items, raters and testing occasions. There is also strong evidence of validity: the CAPS has excellent (>0.90) convergent and discriminant validity, diagnostic utility, and sensitivity to clinical change.

The Posttraumatic Stress Symptom Scale, Self-Report (PSS-SR) [77] is administered to assess self-reported severity of PTSD symptoms [78]. The PSS-SR consists of 17 items corresponding to the 17 diagnostic DSM-IV-TR criteria of PTSD which are rated on a 3-point Likert scale, where 0 = not at all, 1 = a little bit, 2 = somewhat and 3 = very much. It yields a total score measuring symptom severity (range 0 to 51), as well as separate severity scores for re-experiencing (range 0 to 15), avoidance (range 0 to 21), and arousal (range 0 to 15). The PSS-SR is assessed before each treatment session, to assess changes in the PTSD symptoms during treatment.

There is a satisfactory internal consistency (Cronbach’s alpha = 0.91), high test–retest reliability (*r* = 0.74) and good concurrent validity (sensitivity = 62%, positive predictive power = 100%, negative predictive power = 82% [77,79]. In a sample of patients with a first-episode psychosis the screening performance of the PSS-SR was tested against CAPS interview results [80]. The data suggest that the PSS-SR can be a useful screening instrument for PTSD in this group of patients. Sensitivity was 0.83 and specificity was 0.83.

The T.TIP Adverse Events Questionnaire (T-AEQ) is a checklist that establishes treatment safety. In seven questions the patient is asked to report any self-inflicted pain or injury, suicide attempt, deliberately hurting or physically wounding another person, excessive use of alcohol, excessive use of drugs, having needed help because of a crisis and being admitted to a psychiatric hospital. The time frame is two months.

The Adverse Events session ratings assesses adverse events in the participant before and after treatment sessions (sessions two and three), for example, being suicidal, hearing voices. Participants respond on a 10-point visual analog scale (VAS;‘no, not at all’ to ‘yes, very much’) to the questions.

### Measurements – effects of treatment on psychopathology and cost effectiveness

See Table 2, Secondary outcome measurements.

**Table 1:** Measurements primary objectives: PTSD and safety of treatment

**Primary outcome**	**MeasurementInterview(i)**, **self-report(s)**	**T0 baseline**	**T1 midtreatment**	**Within-session (1–8)**	**T2 posttreatment**	**T6 FU 6 months**	**T12 FU 12 months**
PTSD	CAPS (i)	x			x	x	x
	PSS-SR (s)	x		1, 2, 3, 4, 5, 6, 7, 8	x	x	x
Adverse events	TAEQ (s)	x	x		x	x	x
	AE session ratings (s)			2 ,3			

*AE* adverse events, *CAPS* Clinician Administered Post traumatic stress disorder Scale, *FU* follow up, *PSS-SR* Posttraumatic Stress Disorder Symptom Scale - Self Report, *TAEQ* T.TIP Adverse Events Questionnaire.

**Table 2:** Measurements secondary objectives: psychopathology and cost effectiveness

**Secondary outcome**	**MeasurementInterview(i),self-report(s)**	**T0 baseline**	**T1 midtreatment**	**T2 posttreatment**	**T6 FU 6 month**	**T12 FU 12 months**
Paranoid thinking	GPTS (s)	x		x	x	x
Verbal hallucinations	AHRS (i)	x		x	x	x
Delusions	DRS (i)	x		x	x	x
Basic assumptions	AVH-BAS (s)	x	x	x	x	x
Verbal hallucinations	ESM (s, psymate)	x		x		
Depression	BDI-II (s)	x		x	x	x
Social functioning	PSP (i)	x		x	x	x
Posttrauma cognitions	PTCI (s)	x	x	x	x	x
Remission	SCI-SR (i)	x		x	x	x
Care consumption	TiC-P (s)	x		x	x	x
Health outcome	EQ-5D (s)	x		x	x	x

*AHRS* Auditory Hallucination Rating Scale, *AVH-BAS* Auditory Verbal Hallucinations – Basic Assumptions Scale, *BDI-II* Beck Depression Inventory - II, *DRS* Delusion Rating Scale, *ESM* Experience Sampling Method, *EQ-5D™* EuroQol - 5 dimensions *GPTS* Green ParanoiaThought Scale, *PSP* Social Performance Scale *PTCI* Posttraumatic Cognitions Inventory, *SCI-SR* Structured Clinical Interview for Symptoms of Remission for the PANSS, *TiC-P* Trimbos/iMTA questionnaire for Costs associated with Psychiatric Illness - short version.

The Green Paranoid Thought Scales (GPTS) [81] is a self-report measure that assesses changes in paranoid experiences and in ideas of reference. It consists of 32 statements about experiences in the last month, sixteen items about ideas of persecution and sixteen items about ideas of reference. Items are scored on a Likert scale ranging from 1 (not at all) to 5 (totally). The range of the total score is 32 to 160, with higher scores indicating higher levels of paranoia. The GPTS has good internal consistency, is valid, reliable and sensitive to clinical change [81].

The Auditory Hallucination Rating Scale (AHRS), which is part of the Psychotic Symptom Rating Scale(PSYRATS) [82], is an eleven questions interview that assesses the severity of auditory hallucinations: their frequency, duration, location, loudness, causal attribution, negative content, the severity of negative content, the extent and the severity of discomfort and suffering, the disruption of daily life because of voice hearing and the experience of control over voices. Scores are on a range from 0 (for example,‘not’) to 5 (for example,‘continuously’). The range of the total score is 11 to 55. The inter-rater reliability is excellent (.79 to 1.00).

The Delusion Rating Scale (DRS) is part the Psychotic Symptom Rating Scale (PSYRATS) [82]. It assesses the severity of delusions. Six questions go into the extent and the duration of preoccupation with the delusion, the credibility, the extent and severity of discomfort and suffering, and the disruption of daily life because of the delusions. Items are scored from 0 (for example, ‘not’) to 5 (for example,‘continuously’). Total score ranges from 0 to 30. Inter-rater reliability is excellent (.79 to 1.00).

The ESM is an assessment technique designed to obtain repeated self-reports, using an electronic beeper that signals subjects to fill in a questionnaire at preselected but randomized time points for several days [83]. In T.TIP the effect of treatment on auditory (verbal) hallucinations is studied. Therefore, only participants that experience verbal hallucinations every day may participate. Participants who will be part of this ESM study carry a palmtop-like device called a Psymate[84], programmed to beep 10 times a day for six consecutive days at T0 and T6. The questionnaire consists of 50 self-exploratory questions used in previous studies, beginning with the most transient experiences (mood, thoughts and symptoms), followed by more stable items (context) and retrospective items in last position. All items are scored on Likert scales (range 1 to 7) or category boxes.

ESM has been proven to be a valid, reliable and feasible method of investigating psychotic experiences [35,85] by the use of face validity, comparison of aggregated data between groups, correlations between similar and dissimilar items and determining associations with available behavioral/external referents. ESM is useful for refining the phenomenology of psychotic symptoms, for investigating etiological mechanisms underlying psychosis and in clinical practice [86,87].

The Beck Depression Inventory second edition (BDI-II) [88] is a self-report that consists of twenty-one items. Each item is rated on a 4-point Likert-type scale ranging from 0 to 3, with higher scores indicating higher levels of depression. The measure asks respondents to rate statements characterizing how they have been feeling throughout the past two weeks. The maximum total score for all 21 items is 63. Score categories range from 0 to 13 (minimal depression), 14 to 19 (mild depression), 20 to 28 (moderate depression) and 29 to 63 (severe depression). Good psychometric properties have been shown for the original BDI [89] and for the 1996 revision BDI-II [88] that is adapted to depression criteria in the DSM-IV. The BDI-II shows good validity compared to the Hamilton Depression Rating Scale (Pearson *r* of 0.71). The test has high internal consistency (*α*=.91) [62].

The Personal and Social Performance Scale (PSP) [90] assesses social functioning as a secondary treatment outcome measure. The PSP scale is a clinician-rated scale that measures personal and social functioning in the domains of socially useful activities (for example, work and study), personal and social relationships, self-care, and disturbing and aggressive behaviors. Difficulty in each area is rated according to set criteria, using a six-point scale: absent, mild, manifest but not marked, marked, severe, and very severe. On the basis of the subscale ratings, a global score is rated by the interviewer, ranging from 1 to 100 in ten point intervals, where lower scores indicate lower functioning.

The PSP was shown to be able to detect changes in patients with both stable and acute psychotic disorders [91]. The PSP has been developed through focus groups and reliability studies on the basis of the social functioning component of the DSM-IV-TR Social and Occupational Functioning Assessment Scale (SOFAS). The PSP showed good (>0.80) interrater reliability and test–retest reliability [90-92].

The Posttraumatic Cognitions Inventory (PTCI) [93] assesses cognitions after having experienced a trauma. Note that the PTCI will be mentioned again in the section on ‘Tertiary measurements: predictors, moderators and mediators of treatment outcome’. The PTCI serves two goals: (a) post traumatic cognitions may be altered as a consequence of treatment; and (b) post traumatic cognitions may function as a predictor variable of treatment outcome. The PTCI consists of 33 items and three subscales. Negative Cognitions About Self (general negative view of self, permanent change, alienation, hopelessness, self-trust and negative interpretation of symptoms) contains 21 items). Seven items represent the second factor of Negative Cognitions About the World (unsafe world and mistrust of other people). Five items represent the third factor of Self-Blame. Participants rate each item on a 7-point Likert-type scale, from 1 (totally disagree) to 7 (totally agree). Thus, high scale scores indicate stronger maladaptive cognitions. The scores of the three factors are calculated by dividing the sum score by the number of items (factor scores range from 0 to 7). The total score is the sum of the three factor scores (maximum of 21). Internal consistencies of the three subscales were found to be excellent (0.86 to 0.97). The test-retest reliability was good (0.74 to 0.86). The PTCI was shown to discriminate better between individuals with and without PTSD (86% classified correctly) than other tools for assessing trauma-related cognitions [93]. Sensitivity and specificity were proven good [94].

The Structured Clinical Interview for Symptoms of Remission for the PANSS (SCI-SR, eight items) [95] is a brief eight-item interview that is used in research and treatment settings to assess remission of psychotic symptoms, based on eight items of the Positive And Negative Syndrome Scale (PANSS) [96]. In the present study it is part of the research of economic costs. The participant has to confirm functional status on four items: delusions, unusual content of thought, hallucinations and apathy. Scores on the other four items are based on observation and participants’ responses: conceptual disorganization, lack of spontaneity, blunted affect and posturing. Each item is rated using criteria described in the PANSS Manual. The PANSS determines severity of symptoms on 7-point Likert scales. Remission on the PANSS-SCI-SR is defined as a score of three (mild) or less for each item, maintained over a six-month period [97]. Compared to the total PANSS score the specificity of the remission criteria was 85%. The sensitivity was 75%. The remission criteria are both sensitive and specific indicators of clinical status. Additional analyses are required to determine if remission status predicts other outcomes, such as employment, independent living, and prognosis [98].

The Trimbos/iMTA questionnaire for Costs associated with Psychiatric Illness (TiC-P, short version) [99] will be used to assess the costs. Trimbos and the Institute for Medical Technology Assessment (MTA) developed this care consumption list. The TiC-P is commonly applied in the economic evaluation of treatment in mental health care, including trials on the cost utility of brief psychological treatment for anxiety (for example, see [98,100]. To be able to calculate the total direct medical costs, the first part of the Tic-P assesses the total number of medical contacts (outpatient visits, length of stay in hospital, use of medication, and so on). Afterwards, the data will be multiplied by unit costs of the corresponding health care services. Reference unit prices of health care services will be applied and adjusted to the year of this study according to the consumer price index. In the second part of the TiC-P a short form of the Health and Labor questionnaire (HLQ) [101] will be used to collect data on productivity losses. The Short-Form HLQ (SF-HLQ) consists of three modules that measure productivity losses: absence from work, reduced efficiency at work and difficulties with job performance.

The EuroQol (EQ-5D™) [102,103] is a standardized instrument for measuring health outcome. This patient-reported outcome measure has five dimensions: mobility, self-care, usual activities, pain/discomfort and anxiety/depression, and each dimension has three levels (no problem, some problem, severe problem). Together, these five dimensions make a simple descriptive profile that can be compared to a total of 243 health states scored using values obtained from a survey of the general population. Participants also point out a single index value for current health status on a scale from 0 (‘worst possible health’) to 100 (‘best possible health’). The EQ-5D measures and expresses quality of life in utilities. Utilities can be combined with cost-effectiveness data into Quality Adjusted Life Years (QALYs). The EQ-5D has good reliability and validity [103].

### Measurements – links between trauma exposure, PTSD and psychosis

See Table 3, Tertiary outcome measurements. Note that the measurements of psychosis mentioned in Table 2 are necessary, too, for examination of the tertiary objectives.

T.TIP screener*,* including the TSQ*.* The T.TIP screener is designed for the purpose of briefly asking: a) about having experienced overwhelming or life threatening experiences (‘yes’ or ‘no’); and b) If ‘yes’, then participants are asked what kind of PTSD DSM-IV-TR criterion A traumatic events they have experienced: physical abuse, sexual abuse, emotional abuse, extreme neglect and/or accidents/disasters/war. Because patients in this specific patient population may also have experienced traumatic events during psychotic episodes, this category was added. For each category of traumatic events the participant chooses between ‘yes, one traumatic experience’, ‘yes, more than one traumatic experience’ or ‘no traumatic experience’. c) To gain more information about the phenomenological similarities between trauma content and content of the symptoms of their psychosis, participants are asked about similarities (‘no similarity at all’, ‘some similarity’ or ‘strong similarity’). d) Participants then answer questions about PTSD symptoms they may suffer, using the TSQ [36]. The TSQ is a 10-item scale with the five re-experiencing items and five hyperarousal items of the PSS-SR. All items are answered with binary ‘yes' (symptom is present two times a week or more) or 'no’ (symptom is not present or present once a week) responses; the minimum score is zero and the maximum score is ten. The TSQ has a good sensitivity in assessing potential PTSD in crime victims (0.76) and rail crash victims (0.86), as well as a high specificity in both of these groups (0.93 and 0.97, respectively) [36]. The TSQ has also shown to be a good measure to predict future PTSD in assault victims, with a sensitivity of 0.85, a specificity of 0.89, and an efficiency of 0.90 [104]. Both studies mentioned above [36,104] calculated that the optimum cut-off score is 6; any combination of six or more re-experiencing or hyper arousal symptoms predicts PTSD best. The TSQ is not yet calibrated for people with psychotic disorders.

**Table 3:** Measurements tertiary objectives: links between trauma exposure, PTSD and psychosis

**Tertiary outcome**	**MeasurementInterview(i)self-report(s)**	**Screening**	**T0 baseline**
Trauma categories; PTSD risk	T.TIP screener /TSQ (s)	x	
Traumatic events	ITEC (i)		x

*ITEC* Interview for Traumatic Events in Childhood, *T.TIP screening/TSQ* TTIP screening including the Trauma Screening Questionnaire.

The Inventory of Traumatic Events in Childhood (ITEC) [65] is used to interview the participant for traumatic events in his or her life. The ITEC consists of five subcategories: sexual abuse, physical abuse, emotional or psychological abuse, emotional and physical neglect, and ‘other traumas’ that include items such as disaster, war, accidents, illness and death of loved ones. Within each category (for example, physical abuse) a number of specific events are presented (for example,‘You were beaten or got punched’). The participant has to respond to several questions: ‘Did this ever happen to you? ‘, ‘Who did this to you?’, ‘How old were you when this happened?’, ‘Did this occur once, or more?’

For the purpose of the study (a) a category of traumatic events during psychotic episodes was added. This category includes specific events such as ‘A psychosis in which you hurt yourself or tried to kill yourself’; ‘a psychosis in which you were locked up or tied up against your will?’; and b) an assessment was added of both intensity (SUD) and frequency of intrusions for each reported traumatic event. The psychometric properties are good [65]. The scales had good internal consistency (>0.70), except for the physical neglect subscale and an excellent (>0.90) inter-rater reliability. The scales were highly associated with equivalent scales of the Childhood Trauma Questionnaire (CTQ) (that is, good convergent validity) and showed good correspondence with patient file information (that is, good criterion validity). Internal reliability of the ITEC subscales range from .58 to .89 with a mean of .79. The reliability for the physical neglect scale is inadequate, while the other scales display moderate to good reliability. Results showed excellent agreement between the raters for most subscales (intraclass correlation coefficient (ICC) sexual and physical abuse = 1.00; ICC emotional abuse and neglect = .99; ICC witnessing physical abuse = .88; ICC witnessing emotional abuse = .96) and good agreement for the physical neglect scale (ICC = .72). Additionally, high correlations with the corresponding subscales of the CTQ [105-107] were obtained, indicating good convergent validity. Finally, criterion validity was assessed by comparing the presence of maltreatment as mapped by the ITEC with patient file information. Data indicated that the ITEC's sensitivity was excellent, and their scores on their parallel ITEC subscales uniquely predicted sexual and physical abuse and neglect. This was not the case for emotional abuse.

### Measurement – moderators, mediators and predictors of treatment outcome

See Table 4, Quaternary measurements. Note that only measurements on participant variables are included. Look at the therapist ratings section for explanation of measurements of therapist variables.

The Bullying Questionnaire (BQ) [108,109] is used to collect information about being severely bullied in childhood (age <17) as a predictor variable of treatment outcome. The BQ is derived from the original Olweus Bully/Victim list, which has 40 items. The BQ has five items. The BQ questions have been used in the European network of national schizophrenia networks studying Gene-Environment Interactions (EU-GEI). The BQ questions are taken from the Environmental Risk (E-Risk) longitudinal twin study which they adapted from Olweus and have used in several publications, showing good test-retest reliability [108] and reasonable inter-raterreliability [110].

Participants answer if they have been bullied by other children, if they have been physically hurt or injured by other children, if they have been hurt emotionally or psychologically, how severe the bullying was, and if they ever have bullied other children themselves. Scores are on a range from zero (‘never’ or ‘not’) to four (‘often’ or ‘severe’) and total scores of the BQ thus range from 0 to 20.

The Tonic Immobility Scale–Adult Form, Part 1- Short Version(TIS-A-1-SF) [111]) is used to study tonic immobility as a predictor of treatment outcome. TI as a reaction to a traumatic event is originally found mostly in sexually abused women [112] but is also known to occur during other traumatic events. The original TIS-A-Part 1 addresses symptoms of tonic immobility and peritraumatic fear and perceived inescapability [113]. Thus, this TIS-A-Part 1 score represents the individual’s experience of various aspects of the TI response during the traumatic event. The TIS-A-Part 1-Short Form specifically addresses TI. It consists of four items, assessing four possible responses during the traumatic event: (1) freezing, (2) immobility, (3) not being able to shout or scream and (4) possibility of escaping the situation. Answers are on a 7-point Likert-type scale (0 to 6). Psychometric properties of the original TIS-A scale [113] were good, but no data are available with regard to the short version used in the T.TIP study.

**Table 4:** Measurements quaternary objectives: predictors, moderators, mediators (participant variables)

**Quaternary outcome**	**MeasurementInterview(i)self-report(s)**	**T0 baseline**	**T1 midtreatment**	**Within-session (1 to 8)**	**T2 posttreatment**	**T6 FU 6 month**	**T12 FU 12 months**
Bullying	BQ (s)	x					
Tonic immobility	TIS-A-1-SF (s)	x					
Working memory	RIR	x					
Credibility participant	CS-PF (s)			1, 8			
Burden participant	BS-PF (s)			1, 8			
Harm expectancy pre	session rating (s)			2, 3			
Harm experience post	session rating (s)			2, 3			
Demographics	TDQ	x					
Self esteem	SERS-SF (s)	x	x		x		
Illness beliefs	PBIQ (s)	x	x		x		
Post trauma cognitions	PTCI (s)	x	x		x	x	x
Cognitive bias	DACOBS (s)	x	x		x		
Memory	MCQ-RF (s)			2, 3, 8			
Social support	MOS-SSS (s)				x		

*AVH-BAS* Auditory Verbal Hallucinations Basic Assumptions Scale, *BQ* Bullying Questionnaire, *BS-PF* Burden Scale – Participant Form, *DACOBS* Davos Assessment of Cognitive Biases Scale, *FU* follow up, *MCQ-RF* Memory Characteristics Questionnaire – Revised Form (adapted for T.TIP), *MOS-SSS* Medical Outcome Studies - Social Support Survey, *PBIQ* Personal Beliefs about Illness Questionnaire – Revised version, *PTCL* Posttraumatic Cognitions Inventory, *RIR* Random Interval Repetition, *SERS-SF* Self Esteem Rating Scale - Short Form, *TDQ* T.TIP demographic questionnaire, *TIS-A-1-SF* Tonic Immobility Scale- Adult Form-Part 1-Short Form.

The auditory Random Interval Repetition (RIR) task is a computerized reaction time task, that is studied as a possible predictor and moderator of treatment outcome. During this task participants wear headphones that present repetitive beeps of 200 Hz, each lasting for 50 ms. The inter-stimulus-interval (ISI) on this computerized RIR task is either 850 or 1450 ms. Participants are instructed to respond as fast as possible by pressing a bar, every time they hear a beep, and the reaction time (RT) is the dependent variable. The RIR task is carried out under two conditions of three minutes each: 1) while making a distracting task, in this case eye movements, elicited by the index-finger of the research assistant, moving horizontally at 30 cm from the eyes at a pace of two seconds per cycle, and 2) without a distracting task. The order of the conditions is counterbalanced to control for carry over effects. The degree to which eye movements tax the central executive component of the working memory is operationalized as the slowing down of RTs and reduced accuracy (more errors and non-responses) during the distracting task compared with the no-distracting task condition. The task is constructed and performances measured with E-Prime 1.2 software.

To have a RT task carried out while the subject is being distracted simultaneously, is a valid way to assess the presence and severity of cognitive taxing [114]. RTs to auditory cues presented at an RIR task, provide a valid and highly sensitive measure of taxation of the central executive component of the working memory [115]. Laboratory experiments with undergraduates have shown that 1) making eye movements during a stimulus discrimination task slows down RTs and raises the number of errors made [116] and 2) more taxing of the working memory results in higher RTs, more errors and more non-responses [117]. In a pilot study with 12 patients with a psychotic disorder we found the same patterns in the performance on the RIR task with three conditions: no eye movements, eye movements at one second per cycle and eye movements at two seconds per cycle (publication in preparation). In T.TIP two seconds per cycle will be used.

Several measures of treatment credibility, burden, harm expectancy and harm experience are administered to both therapists and participants.

First, the T.TIP Therapist attitude questionnaire (T-TAQ) aims to measure therapist attitude towards the treatments (PE and EMDR). This has two goals: (i) to study therapist treatment attitude as a predictor variable on treatment outcome, and (ii) to study the impact of training and experience on therapist treatment attitude. The T-TAQ is repeatedly administered in the context of training and supervision sessions. The T-TAQ consists of 15 items, divided into three parts a, b (with within session extension) and c (with within session extension). (a) The Impact of Training and Therapeutic Experience Questionnaire – therapist form (ITTEQ-TF) assesses the therapist factor of ‘Training and therapeutic experience’ in PE and EMDR. In this three item therapist self-report list, therapists disclose on a 1 (‘totally disagree’) to 10 (‘totally agree’) VAS their level of (1) training in EMDR/PE, (2) experience in carrying out EMDR/PE and (3) the number of patients treated successfully using EMDR/PE. (b-1)The Credibility Scale- therapist form (CS-TF). Therapists respond to five treatment credibility statements regarding PE and EMDR on a 1 to 10 VAS: (1) This treatment seems logical to me. (2) This treatment seems scientific to me. (3) If I have a PTSD, I would choose this treatment. (4) This treatment would be effective for most people. (5) If a close friend or relative has PTSD, I would recommend this therapy to them. The CS-TF is an adaptation of the original Credibility/Expectancy Questionnaire [118] which demonstrated high internal consistency within each factor (credibility and expectancy) and good test–retest reliability. (b-2) The Credibility Scale- therapist session form (CS-TSF) is an adaptation of the CS-TF. It is an extra 10-item credibility rating that will be applied within actual treatment sessions (1 and 8); the therapist rates not only his or her own opinions on credibility, but in addition makes an estimation of how he or she expects the participant to rate the credibility of the treatment on the same five questions of the CS-TF ('logical' and so on). (c-1)The Burden Scale-therapist form (BS-TF). Seven questions regarding PE and EMDR assess ‘Perceived barriers’ on a 1 to 10 VAS: (1) PTSD will get worse, (2) psychosis will get worse, (3) other comorbid symptoms will get worse, (4) the treatment is a burden to the patient, (5) the treatment is a burden to the therapist, (6) the treatment will facilitate drop out, (7) the treatment will facilitate crisis or admissions to hospital. The BS-TF is inspired by studies that compare the burden and endorsement of CBT and EMDR (for example, studies using the Distress/Endorsement Validation Scale (DEVS)) [119,120]. The BS-TF is not an adaptation of the DEVS, however. It is adapted to suit the research questions of the study regarding (perceived) barriers for treatment in the population of patients with psychosis. There are no psychometric evaluations available. (c-2) The Burden Scale- therapist session form (BS-TSF) is an adaptation of the BS-TF. It is an extra three-item burden rating that will be applied within actual treatment sessions (1 and 8); the content is limited to estimations by the therapist of how he or she expects the participant to rate the burden of the treatment on a 1 to 10 VAS (‘no, not at all’ to ‘yes, very much’): (1) Does the participant fear treatment?, (2) Is he or she hesitant to have this treatment?, and (3; only for PE) Does the participant expect that listening to the recordings at home will be a burden?

The Treatment Preference and Experience-Therapist Scale (TPE-TS) allows therapists, before treatment starts, to score their treatment experience with both PE and EMDR and their personal preference to start with PE and EMDR with this particular patient, on a VAS-scale from 0 to 100. Post treatment the therapist again scores his/her personal preference to use PE and EMDR with this particular patient using the same scale. Psychometric evaluations are not available.

The Burden Scale – Participant Form (BS-PF) assesses burden in participants before (expected) and after (experienced) treatment in both conditions, PE and EMDR. Before treatment the participants responds on a 10-point Likert-type Scale (‘no, not at all’ to ‘yes, very much’) to the following questions: (1) Do you fear treatment?, (2) Are you hesitant to have this treatment?, and (3, only for PE) Do you expect that listening to the recordings at home will be a burden? After treatment the participant responds on a 10-point VAS (‘no, not at all’ to ‘yes, very much’) to the questions: (1) Do you feel that afterwards the treatment was less burdensome than you had expected?, (2) Was the treatment much of a burden to you?, (3) How much of a burden was it to listen to recordings at home? No psychometric evaluations are available.

The Credibility Scale - Participant Form (CS–PF) assesses credibility before and after treatment in both conditions PE and EMDR. Participants respond on a 10-point VAS (‘no, not at all’ to ‘yes, very much’) to the statements: (1) This treatment seems logical to me, (2) This treatment seems scientific to me, (3) If I have a PTSD, I think this treatment will help me, and (4) If a close friend or relative has PTSD, I would recommend this therapy to them. There are no psychometric evaluations available.

The Harm Expectancy session ratings assess the treatment harm expectancies (before the session) and experienced harm (after the session) in two sessions (sessions 2 and 3). Participants respond on a 10-point VAS (‘no, not at all’ to ‘yes, very much’) to the questions, describing expected or actually experienced fear of going crazy, panicking and losing control, panicking and drop out of the session, or yet another harm expectancy or harm experience.

The T.TIP Demographic questionnaire (TDQ) assesses basic personal, social and medical data as predictors of treatment outcome variables: 1) age; 2) date of birth; 3) country of birth (participant, father, mother); 4) highest level of achievement in education; 5) daily housing/living situation; 6) DSM-IV-TR diagnoses; 7) substance abuse or dependence; 8) current medication; 9) years of illness of psychosis and PTSD.

The Self-Esteem Rating Scale - Short form(SERS-SF) [121] assesses the mediator/moderator variable of self esteem on treatment outcome. The 20-item SERS-SF, with its positive and negative self-esteem subscales, appears to be a valid and reliable self-esteem measure for individuals with severe mental health problems. The original 40-item SERS was reduced to a psychometrically good 20 item version of the SERS, the SERS-SF. The SERS-SF [121] has 10 positive and 10 negative items on self-esteem. The two scales have excellent internal consistency (*α* = .91 positive scale; *α* = .87 negative scale). The test–retest reliability of both scales is high (r =0.90; r =0.91). The SERS total score correlated highly with both scales (r = 0.72 and r = 0.79), indicating good convergent validity.

The Personal Beliefs about Illness Questionnaire – Revised version (PBIQ-R*)*[51] assesses the mediator/moderator variable of cognitions about, and coping with, being ill on treatment outcome. The original PBIQ was a 16-item scale developed to assess people’s appraisals of a psychotic illness in five domains, namely (1) control over illness; (2) self as illness; (3) illness as an impediment to the attainment of goals; (4) humiliation and guilt, and (5) need for social containment. A revised 29-item scale (PBIQ-R) measures five different modes of experience following a psychotic illness: shame (six items); loss (seven items); entrapment in psychosis (six items); control over illness (five items); and social marginalization/group fit (five items). Participants respond to statements (for example, ‘I will always need medical care’) by choosing between: ‘strongly disagree’, ‘diagree’, ‘agree’ or ‘strongly agree’. The PBIQ has no total score. The PBIQ-R has good predictive validity for relapse as a result of negative appraisals of illness and coping skills [122].

The Posttraumatic Cognitions Inventory (PTCI) [93] is assessed as a mediator/moderator variable on treatment outcome. For a description, see the section on Measurements – effects of treatment on psychopathology and cost effectiveness.

The Davos Assessment of Cognitive Biases Scales (DACOBS) [123] assesses the mediator/moderator variable of cognitive biases in psychoses on treatment outcome. Cognitive problems and biases play an important role in the development and continuation of psychosis. The DACOBS has been developed as a self-report measure of these deficits and processes. It assesses seven statistically independent deficient thought processes. Each factor is represented with six items: jumping to conclusions, confirmation bias/dogmatism, selective attention for threat, self as target, theory of mind problems, subjective cognitive failure and avoidance behavior. Each item is a statement that is scored on a 7-point Likert scale within a two-week time frame. Reliability was good (*α* = 0.90; split-half reliability = 0.92; test-retest reliability = 0.86). The DACOBS distinguishes between schizophrenia spectrum patients and normal control subjects. It is reliable and valid and may be used in research.

The Auditory Verbal Hallucinations – Basic Assumptions Scale *(*AVH-BAS*;* van den Berg *etal*., publication in preparation) assesses the mediator/moderator variable of participants' basic assumptions about voice hearing on treatment outcome. The AVH-BAS is especially developed for this RCT. In another study outside of T.TIP responses of 176 voice-hearing patients were assessed on several items that reflect cognitive and emotional appraisal of voices. After item- and factor-analysis, 14 AVH-BAS-items remained that assess cognitive assumptions about voices. Four factors have shown to be relevant: (1) negative self-esteem, (2) guilt, (3) powerlessness and (4) danger/threat. Initial analyses suggest that the AVH-BAS may be a reliable and valid instrument.

The Memory Characteristics Questionnaire – Revised Form (MCQ-RF) [119], which was adapted for the present study, assesses aspects of the traumatic memory as a mediator/moderator variable and its influence on treatment outcome. The MCQ-RF is administered within treatment sessions. The MCQ-RF assesses self-reported characteristics of trauma memories. The original MCQ has a test-retest reliability total scale of r =. 86. In T.TIP we used a shortened version (three items) of the re-experiencing subscale (test-retest reliability r = .86): sense of reliving, sense of here and now, and perceptual elements. We added one item about memory-related emotions (for example, dissociation, anxiety, anger, guilt and depression) and one item about memory-related image characteristics (for example, color, light and movement). Participants were asked to rate their trauma memories for each of the items on a 0 to 100 scale.

The Medical Outcome Studies - Social Support Survey (MOS-SSS) [120] assesses experienced social support as a possible predictor variable of treatment outcome. The survey consists of four separate social support subscales and an overall functional social support index. A higher score for an individual scale or for the overall support index indicates more support. Overall index internal-consistency reliability: α=0.97, one-year test-retest stability= 0.78. For our purposes only the subscales ‘emotional support’ (ES) and ‘instrumental support’ (IS) are administered to the participants. On a Likert type scale from 1 (‘none of the time’) to 5 (‘All of the time’) participants respond to four ES-items: (1) Someone you can count on to listen to you when you need to talk, (2) Someone to give you information to help you understand a situation, (3) Someone to give you good advice about a crisis, (4) Someone to confide in or talk to about yourself or your problems, and to four IS-items (5) Someone whose advice you really want, (6) Someone to share your most private worries and fears with, (7) Someone to turn to for suggestions about how to deal with a personal problem and (8) Someone who understands your problems.

The internal-consistency reliability and one-year stability for subscales ES and IS: α=0.96, stability =0.72 [120].

### Fidelity checks

The interrater reliability of interview assessments with the CAPS, PSP and SCI-SR remission tool is accounted for ingroup wise sessions for interrater reliability enhancement and interrater reliability measurements. Interrater reliability measurements will be carried out monthly, by presenting cases to the research assistants for scoring. All assessments that research assistants will perform at T0, T2, T6 and T12 are presented in a written report, to be reviewed by the researchers.

Therapists receive four days of training in each treatment protocol, that is, PE and EMDR. All treatment sessions will be videotaped. A selection will be rated for treatment fidelity. All therapists will be supervised by highly skilled professionals (AvM, AdJ and CdR, respectively) to evaluate, guide and approve the case conceptualizations and treatment interventions. Monthly, four-hour group supervision supports the therapist for the whole duration of the experimental intervention.

Every session the therapist will fill out a self-report questionnaire about the elements and the steps in the treatment protocol. Deviations from the protocol will be reported to the supervisor.

### Therapist ratings

During the whole process of training and treatment, assessments will be carried out of therapists' opinions and expectations regarding the credibility and burden of treatment. As indicated in the Instruments section the T-TAQ is administered, comprising the ITTEQ-TF*,* the CS-TF and the BS-TF. The ITTEQ-TF is integrated into the training and supervision on PE and EMDR and is rated at different levels: before treatment training ('prior'), halfway through training ('theory'), after training ('first practice'), in therapist group supervision session number four ('novice'), at the end of the supervision series ('competent') and at the half year follow up ('expert'). The session versions of both the CS-TF and BS-TF (CS-TSF and BS-TSF) will be used additionally within treatment sessions one and eight.

The TPE-TS is administered to therapists before and after treatment of each participant.

### Analyses

The primary outcome on the PTSD measures will be analyzed using Linear Mixed Models (LMM) with the baseline value as a covariate. LMM will also be used with the secondary outcome measures. Sensitivity and specificity of the screener will be calculated with Receiver Operating Characteristic curves. The predictors will be assessed with (logistic) regression analysis. The moderator and mediator analyses will be calculated with 5,000 bootstraps [124]. The association between working memory and the outcome of therapy is performed by analysis of covariance (ANCOVA’s) and multiple regression analysis. The ESM data will be analyzed by multilevel linear regression modeling using STATA. In case of an effective treatment a cost-effectiveness analysis will be performed. The incremental cost-effectiveness ratios (ICERs) are considered to be a single-point estimate of an underlying continuum. Acceptability curves are produced with bootstrap simulations and confidence intervals. The outcome will be costs in Euro’s per QALY and the costs in Euro’s per day without PTSD gained. If no intervention is effective, which is not expected, a cost-minimization calculation will be done.

## Discussion

This present so called ‘T.TIP trial’ has several strengths that have the potential of making the T.TIP trial a forerunning experiment.

First, it is hypothesized that the T.TIP trial will make it possible to calibrate an efficacious and efficient short screening tool for assessing PTSD in the group of patients with psychosis. Given the fact that PTSD is underdiagnosed in this patient population [19,20], an instrument that quickly, safely and accurately assesses the risk of PTSD may be a significant aid to clinical practice.

The main goal of the study is to treat PTSD in patients with psychosis. The comparison of two trauma-focused treatments to a waiting list condition will provide answers about the effectiveness of these trauma-focused treatments on PTSD symptoms. This is the first controlled study that directly assesses the effectiveness of two guideline-recommended trauma-focused treatments in this severely mentally ill population that is generally excluded from clinical trials [29]. Further, it is a large study, including enough patients to make powerful conclusions. This would be an important contribution to treatment options for people suffering from psychosis and PTSD, as PTSD symptoms negatively influence the level of functioning and quality of life in patients with psychosis [9,10]. Conceivably, reducing these symptoms will be of great personal value to patients with psychosis who suffer from the consequences of exposure to traumatic events.

What is more, the study is aimed at establishing whether these treatments can be applied safely in this patient population. This is important for the implementation of the treatments, given the fact that clinicians are often hesitant to address issues of exposure to traumatic events in this population because they fear deterioration and crisis [23,24]. Interestingly, in this aspect, we applied short-term (eight sessions) ‘basic’ manualized treatment to this population, without any pre-treatment interventions, such as emotion-regulation, skills training, or stress management. Should these standard trauma-focused treatments prove to be safe, this will strengthen their implementation in clinical practice. A strength of the study is also that it is a multi-center trial, which will enhance the internal and external validity of the interventions.

In addition to PTSD-symptoms and safety, we also studied effects of treatments on psychopathology severity, especially psychotic symptoms. By studying several variables during treatment, we will be able to disentangle the complex interactions between traumatic events, PTSD and psychosis [9-13] and identifying mediators, moderators and predictors of treatment outcome. This is important from a theoretical point of view, but also from a clinical perspective. It will provide practical guidelines for diagnosis and treatment of PTSD in the population of patients with psychosis. One limitation is that only patients are included in the intervention part of the present studywho suffer from psychosis and at the same time meet all PTSD criteria according to the DSM-IV-TR. Should the results on tertiary objectives indicate that there are links between the trauma characteristics and the characteristics of psychosis in this particular group, then these results cannot be generalized automatically to the general population of patients with psychosis (with individuals who have no PTSD symptoms or subthreshold PTSD).

Another important part of the study is the measurement of cost-effectiveness. When PTSD and other symptoms of psychopathology decrease as a result of treatment, it can be expected that patients consume less care, and thereby diminish health costs.

## Trial status

Currently screening and recruiting for participants.

## Abbreviations

AE: adverse events; AHRS: Auditory Hallucination Rating Scale; AVH-BAS: Auditory Verbal Hallucinations Basic Assumptions Scale; BDI-II: Beck Depression Inventory - II; BS-TF: Burden Scale-therapist form; BS-TSF: Burden Scale- therapist session form; BQ: Bullying Questionnaire; BS-PF: Burden Scale – Participant Form; CAPS: Clinician Administered Post traumatic stress disorder Scale; CBT: Cognitive Behavior Therapy; CS-TF: Credibility Scale- therapist form; CS-TSF: Credibility Scale- therapist session form; DACOBS: Davos Assessment of Cognitive Biases Scale; DRS: Delusion Rating Scale; FU: follow up; EMDR: eye movement desensitization and reprocessing; ESM: Experience Sampling Method; EQ-5D™: EuroQol - 5 dimensions; GPTS: Green Paranoia Thought Scale; ITEC: Interview for Traumatic Events in Childhood; ITTEQ-TF: The Impact of Training and Therapeutic Experience Questionnaire – therapist form; MCQ-RF: Memory Characteristics Questionnaire – Revised Form (adapted for T.TIP); M.I.N.I.-plus: M.I.N.I. International Neuropsychiatric Interview-Plus; MOS-SSS: Medical Outcome Studies - Social Support Survey; PBIQ: Personal Beliefs about Illness Questionnaire – Revised version; PE: prolonged exposure; PSP: Social Performance Scale; PSS-SR: Posttraumatic Stress Disorder Symptom Scale - Self Report; PSYRATS: Psychotic Symptom Rating Scale; PTCL: Posttraumatic Cognitions Inventory; RA: Research Assistant; RCT: randomized controlled trial; RIR: Random Interval Repetition; SCI-SR: Structured Clinical Interview for Symptoms of Remission for the PANSS; SERS-SF: Self Esteem Rating Scale - Short Form; SUD: Subjective Unit of Distress; TAEQ: T.TIP Adverse Events Questionnaire; TDQ: T.TIP demographic questionnaire; TF-CBT: trauma focused cognitive behavioral therapy; TiC-P: Trimbos/iMTA questionnaire for Costs associated with Psychiatric Illness - short version; TIS-A-1-SF: Tonic Immobility Scale- Adult Form-Part 1-Short Form; T.TIP screening/TSQ: TTIP screening including the Trauma Screening Questionnaire; VAS: Visual Analogue Scale

## Competing interests

The authors declare that they have no competing interests.

## Authors’ contributions

All authors contribute to both the design and implementation of the study. MvdG is the principal investigator of the study and supervises the development of the study protocol. PdB, DvdB and BvdV are responsible for the logistics within the departments of the psychiatric centers. They take care of organizing and supervising the process of recruitment of participants, training and managing the research assistants, and monitoring and supervising the flow of research reports on assessment and diagnoses. AvM, AdJ and CdR supervise the development of the treatment protocols and the standardized case conceptualization format by PdB, DvdB and BvdV. AvM, AdJ and CdR supervise therapists individually in case conceptualization and in group wise treatment supervision. PdB, DvdB and BvdV prepare the manuals for therapists and research assistants. PdB, DvdB and BvdV recruite and train the research assistants. All authors (PdB, DvdB, BvdV, CdR, CM, EB, AdJ, MvdG and AvM) provide comments on all aspects of the study, and will read and approve the final manuscripts. All authors have read and approved the final version of this manuscript.

## Authors’ information

Paul.A.J.M. de Bont is a clinical psychologist and PhD student at the Radboud University Nijmegen, The Netherlands. He works at GGZ Oost Brabant, Boekel, The Netherlands, in a team for Flexible Assertive Community Treatment for patients with severe mental illness.David P.G. van den Berg is a clinical psychologist and PhD student at the VU University Amsterdam, the Netherlands. He works at the Early Detection and Intervention Team (EDIT) of Parnassia Psychiatric Institute, The Hague, The Netherlands.Berber M. van der Vleugel is a clinical psychologist and PhD student at the VU University Amsterdam, the Netherlands. She works in a Flexible Assertive Community Treatment team for patients with severe mental illness at MHO GGZ Noord-Holland Noord, Alkmaar, The Netherlands.Carlijn de Roos is a clinical psychologist, researcher and an expert on EMDR. She works at MHO Rivierduinen, Leiden, The Netherlands. She is chair of the Dutch EMDR Association.Cornelis L. Mulder is psychiatrist and professor of public mental health at the Department of Psychiatry, Erasmus MC: University Medical Center Rotterdam, The Netherlands.Eni S. Becker is Professor at the Department of Clinical Psychology, Radboud University Nijmegen, The Netherlands. She is chair of "Eperimental Psychopathology and Psychotherapy" of the Behavioural Science Institute, Nijmegen, The Netherlands.Ad de Jongh is professor at the Department of Behavioural Sciences, Academic Centre for Dentistry Amsterdam (ACTA), University of Amsterdam (UvA) and VU University Amsterdam, Amsterdam, The Netherlands. He is honorary professor at the School of Health Sciences at Salford University, Manchester, United Kingdom. He is a board member at the Dutch Foundation of Psychotrauma and the Dutch EMDR Association.Mark van der Gaag is Professor of Clinical Psychology at the VU University Amsterdam and EMGO Institute for Health and Care Research, Department of Clinical Psychology, Amsterdam, the Netherlands. He is Head of Psychosis Research of Parnassia Psychiatric Institute, The Netherlands.Agnes van Minnen is Professor at the Department of Clinical Psychology, Radboud University Nijmegen, The Netherlands. She works as a clinical psychologist and Professor at MHO ‘Pro Persona’, Centre for Anxiety Disorders Overwaal, Nijmegen, The Netherlands.
